# At the Crux of Joint Crosstalk: TGFβ Signaling in the Synovial Joint

**DOI:** 10.1007/s11926-022-01074-6

**Published:** 2022-05-02

**Authors:** Karsyn N. Bailey, Tamara Alliston

**Affiliations:** 1 Department of Orthopaedic Surgery, University of California San Francisco, 513 Parnassus Avenue, CA 94143 San Francisco, USA; 2 UC Berkeley-UCSF Graduate Program in Bioengineering, San Francisco, CA USA

**Keywords:** TGF beta, Joint crosstalk, Osteoarthritis, Subchondral bone, Articular cartilage

## Abstract

**Purpose of Review:**

The effect of the transforming growth factor beta (TGFβ) signaling pathway on joint homeostasis is tissue-specific, non-linear, and context-dependent, representing a unique complexity in targeting TGFβ signaling in joint disease. Here we discuss the variety of mechanisms that TGFβ signaling employs in the synovial joint to maintain healthy joint crosstalk and the ways in which aberrant TGFβ signaling can result in joint degeneration.

**Recent Findings:**

Osteoarthritis (OA) epitomizes a condition of disordered joint crosstalk in which multiple joint tissues degenerate leading to overall joint deterioration. Synovial joint tissues, such as subchondral bone, articular cartilage, and synovium, as well as mesenchymal stem cells, each demonstrate aberrant TGFβ signaling during joint disease, whether by excessive or suppressed signaling, imbalance of canonical and non-canonical signaling, a perturbed mechanical microenvironment, or a distorted response to TGFβ signaling during aging.

**Summary:**

The synovial joint relies upon a sophisticated alliance among each joint tissue to maintain joint homeostasis. The TGFβ signaling pathway is a key regulator of the health of individual joint tissues, and the subsequent interaction among these different joint tissues, also known as joint crosstalk. Dissecting the sophisticated function of TGFβ signaling in the synovial joint is key to therapeutically interrogating the pathway to optimize overall joint health.

## Introduction

The synovial joint consists of multiple tissues, including articular cartilage, subchondral bone, meniscus, and synovium, that interact biologically and mechanically to maintain joint health [1]. During joint degeneration, the disruption of one tissue can lead to overall deterioration by shifting biomechanical loads, increasing inflammation, altering paracrine factors, or inducing aberrant cell-intrinsic signaling. These interactions among joint tissues are referred to as joint crosstalk. Though joint crosstalk is critical for joint health, it can also exacerbate disease. Unravelling the mechanisms and causal relationships among these tissues has been challenging and has limited the development of therapies that modify osteoarthritis (OA) progression.

The transforming growth factor beta (TGFβ) signaling pathway plays an intricate role in maintaining healthy joint crosstalk by carefully regulating homeostasis of each tissue. The delicate balance of TGFβ signaling throughout the joint is non-linear and relies upon control of effector selection, mechanical cues, and spatial localization. Here, we discuss the multifaceted function of TGFβ signaling in joint homeostasis and disease.

## Overview of the TGFβ Signaling Pathway

The TGFβ signaling pathway controls cell behavior through hierarchical, context-dependent, and coordinated regulation [2–4]. The expression pattern of the three TGFβ ligand isoforms, TGFβ1, β2, and β3, is spatially and temporally regulated throughout the lifespan. Each joint tissue expresses ligands capable of activating the TGFβ signaling pathway, as well as the receptors and effectors needed to respond. The expression pattern and relative activity of each component are tightly regulated, which contributes to the diverse effects of TGFβ in the joint.

The TGFβ ligands are secreted as inactive ligands, consisting of the latency-associated peptide and the mature peptide. This complex is then secured to the extracellular matrix (ECM) through latent TGFβ binding proteins (LTBP) and other proteins, such as biglycan, fibrillin, and thrombospondin, many of which have also been implicated in joint homeostasis or disease [5]. Not only do these mechanisms function to locally sequester TGFβ in the ECM, but they also add multiple layers of regulation to TGFβ activation, to be discussed later. Briefly, latent TGFβ is activated through a variety of mechanisms, including those that are sensitive to the mechanical or chemical microenvironment [2]. This ECM-dependent control of TGFβ activity is especially important in the joint. First, tissues with abundant ECM, such as hyaline cartilage and bone, are reservoirs for latent TGFβ. Second, ECM changes that typify joint disease can indirectly disrupt the tight local control of TGFβ sequestration and activity.

Once latent TGFβ ligand becomes activated, it binds to two type II serine/threonine kinase receptors (TβRII), which then recruit and phosphorylate two type I serine/threonine kinase receptors (TβRI) to form a heterotetrameric transmembrane receptor complex [6]. The type I receptors, also termed activin receptor-like kinases (ALKs), calibrate ligand binding affinity and effector selection. Upon ligand binding, the receptors are phosphorylated, which permits effectors to be recruited and activated, including the canonical SMAD2/3 protein and multiple non-canonical effectors, such as SMAD1/5/8, Erk, JNK, Akt, and p38 [5]. Canonically, SMAD2/3-dependent TGFβ signaling is mediated by the ALK5 type I receptor, and SMAD1/5/8-dependent TGFβ signaling is mediated by the ALK1 type I receptor, illustrating one mechanism of effector selection in TGFβ signaling [5]. Once activated, phosphorylated SMAD3 (pSMAD3) forms a complex with the co-Smad SMAD4 and translocates to the nucleus where it binds directly to Smad-binding elements in the promotor, or interacts indirectly with other transcription factors, to alter gene expression [6].

TGFβ signaling exhibits exquisite internal control through negative feedback by a variety of mechanisms. Inhibitory Smads *SMAD6* and *SMAD7* are induced by TGFβ, activin, and BMP signaling and regulate signaling by competitively interfering with the receptor-Smad complex to prevent Smad activation [7]. Additionally, SMAD6 and SMAD7 inhibit TGFβ signaling by recruiting Smurf E3 ubiquitin ligases, SMURF1 and SMURF2, which ubiquitinate the TGFβ receptors and Smads, tagging them for degradation [7]. Multiple other mechanisms of transcriptional and post-transcriptional control, including targeting components of the TGFβ pathway through microRNAs (miRNAs), sumoylation, and regulated localization, also tune the functional activity of this pathway [5, 7]. Another level of complexity in effector regulation is that, in certain contexts, non-canonical effectors can themselves regulate the activity of SMAD2/3, thus impacting downstream signaling and gene expression [2].

Here, we focus on the function of TGFβ in skeletal cells and tissues in joint health and disease. Spatial and temporal control of TGFβ signaling is exerted at multiple levels of the pathway and is context dependent. Disrupting this balance can lead to joint degeneration.

## Overview of Joint Health and Disease

**Fig. 1:**
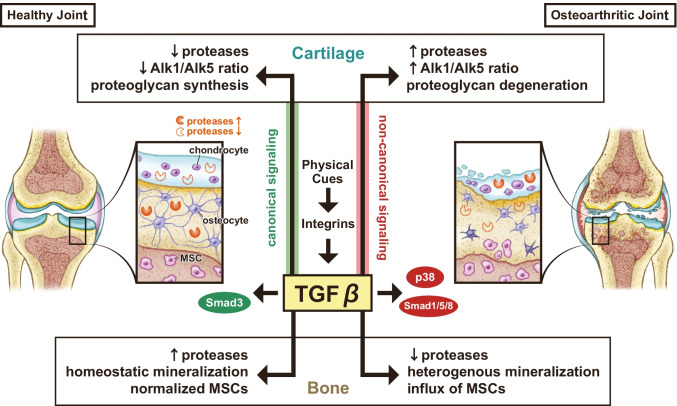
Synovial joint homeostasis relies upon intricate control of TGFβ signaling across multiple joint tissues. Disruption of the complex hierarchical regulation of TGFβ signaling, whether through physical cues, receptor recruitment, or effector selection, impairs synovial joint health and results in joint degeneration in a tissue-specific manner. Proteases, such as MMP13, have differential TGFβ-dependent regulation within each tissue in joint disease, such that elevated proteases in subchondral bone improve bone quality and joint health, yet elevated proteases in cartilage leads to cartilage degeneration

While there are a variety of joint diseases, this review will focus primarily on the impact of TGFβ signaling in OA. OA is a chronic and debilitating joint disease that diminishes mobility and causes severe pain. OA affects 30 million Americans [8] and is a leading cause of disability globally [9]. There is no currently available pharmacologic agent that can delay or prevent the development of OA, and end-stage OA often deteriorates to the point that a joint replacement is necessary. Given the dearth of treatment options for those suffering from OA, there is a pressing need to understand the cellular mechanisms that contribute to joint homeostasis. As mentioned previously, hierarchical regulation of the TGFβ signaling pathway indicates that differential function in various levels of the pathway could result in dysregulated TGFβ signaling (Fig. 1). Importantly, either diminished or excessive TGFβ signaling can perturb joint health.

One genetic syndrome vividly demonstrates the importance of the TGFβ pathway on joint health. Aneurysms osteoarthritis syndrome (AOS) is a familial syndromic form of aortic aneurysm and early-onset OA that is the result of loss-of-function mutations of *SMAD3*. For example, one familial form involves a truncating mutation that removes the MH2 domain, while another familial form involves missense mutations that result in conformational changes of the SMAD3 protein, disrupting protein trimerization [10, 11]. Other musculoskeletal anomalies can also be present in patients with AOS, including intervertebral disc degeneration, osteochondritis dissecans, and meniscal anomalies [11]. Most mutations that lead to AOS are located in the MH2 domain, which is important for SMAD association with TGFβ receptors, SMAD3/SMAD4 oligomerization, and SMAD-dependent transcriptional control [11]. Despite the loss-of-function mutation in *SMAD3*, patients with AOS paradoxically demonstrate increased total levels of SMAD3 protein, excessive activation and nuclear localization of SMAD2 and SMAD3, and higher levels of TGFβ1 in the aortic wall [10]. Taken together, these observations suggest a role for enhanced TGFβ signaling in AOS. While the level of TGFβ and downstream effectors was not assessed in musculoskeletal tissues, it is possible that early-onset OA in these patients coincides with excessive TGFβ signaling in the joint.

Dysregulated TGFβ signaling has been found in various synovial joint tissues in OA. Bone from patients with OA exhibits increased expression of TGFβ1 ligand and *TGFB1* and *SMAD3* gene expression, even at sites distant to joint degeneration [12–14]. Other changes observed in OA bone include altered collagen I production, *RUNX2* expression, osteocalcin, and WNT signaling targets, each of which plays a role in osteoblast differentiation [12, 14, 15]. In human cartilage, *SMAD3* mRNA expression is higher in OA cartilage than in non-arthritic control tissue [16], providing evidence that in some cases, the TGFβ/SMAD3 pathway may be overactive in OA cartilage leading to degeneration. In synovium and synovial fluid from patients with OA, elevated levels of TGFβ1 ligand are a strong predictor of OA progression [17].

Genome-Wide Association Studies (GWAS) provide compelling evidence of the multi-level genetic role of TGFβ signaling in joint disease. Single nucleotide polymorphisms (SNPs) in introns or exons of the *SMAD3* gene have been associated with increased risk of OA [18–21]. More specifically, a missense mutation of an exon of the *SMAD3* gene correlates with increased serum levels of MMP2 and MMP9, enzymes that play a role in articular cartilage degeneration [18]. Genetic variation in intronic SNPs of *SMAD3* also increases risk of hip OA and knee OA, although the specific function of the SNPs is unclear given their intronic location in the *SMAD3* gene [19–21]. In concert with the increased risk of OA with *SMAD3* mutations, *SMAD3* may have a pleiotropic effect on bone mineral density [20]. At the level of the ligand, a SNP within the *LTBP3* gene, encoding a latent TGFβ binding protein that can regulate the availability and activity of TGFβ ligand, was associated with increased risk and clinical severity of hip OA [22]. Noncoding mutations in *GDF5*, a member of the TGFβ super family, or p38 MAP kinase-mediated signaling, a “non-canonical” effector of the TGFβ pathway, are causal variants in OA development [23••]. A single variant within the *TGFB1* gene has been postulated as causal for OA development, a finding that coincides with significant genome-wide enrichment for other genes implicated in TGFβ-signaling, such as *LTBP1*, *LTBP3*, *SMAD3*, and *RUNX2* [24••]. Evidence of a genetic basis for TGFβ mutations in OA development motivates the mechanistic study of TGFβ in joint health to identify appropriate therapeutic targets.

## Mechanoregulation of TGFβ Signaling

At the cellular level, changes in the mechanical microenvironment can alter the effect of TGFβ signaling at different hierarchical levels of the signaling pathway in each joint tissue (Table 1) [25]. Cellular forces resulting from changes in ECM stiffness or cytoskeletal tension can directly activate the TGFβ ligand, rendering it available for downstream signaling [26]. Colocalization of TGFβ receptors TβRI and TβRII to form heteromeric complexes capable of activating downstream signaling is sensitive to cytoskeletal tension in chondrocytes and fluid flow shear stress in osteocytes [27, 28]. Similarly, cytoskeletal tension primes the activity of the chondrogenic transcription factor SOX9 in response to TGFβ or mechanical stimulation [29]. A discrete cell substrate stiffness that is consistent with the physiologic stiffness of healthy articular cartilage stimulates maximal TGFβ-dependent SMAD3 phosphorylation, nuclear localization, and transactivation of chondrogenic genes; substrates that are more or less stiff than healthy articular cartilage diminish the ability of TGFβ signaling to induce chondrogenesis [30].

**Table 1:** Mechanoregulation of TGFβ signaling in the synovial joint

	Reference
Bone	
Colocalization of TβRI and TβRII in osteocytes is sensitive to fluid shear stress	[28]
Loss of osteocytic TβRII impedes subchondral bone response to changes in load	[43•]
Cartilage	
Colocalization of TβRI and TβRII in chondrocytes is sensitive to cytoskeletal tension	[27]
Chondrogenic activation of SOX9 in response to TGFβ is sensitive to cytoskeletal tension	[29]
ECM stiffness affects chondrogenic potential of TGFβ signaling	[30]
Joint motion activates latent TGFβ in superficial zone of articular cartilage	[31]
Cartilage loading reduces *Alk1* and increases *Alk5* mRNA expression	[34, 35]
Aged cartilage has reduced ability to induce canonical TGFβ signaling with load	[38]
Synovium	
Mechanical shearing activates synovial fluid latent TGFβ1	[32]
Mesenchymal stem cells	
Changes in load increases ALK5 in subchondral bone MSCs without increasing ALK1	[39]

Joint loading presents a tissue-level mechanical cue that regulates the function of TGFβ ligand to modulate metabolic activity of chondrocytes. Both the superficial zone chondrocytes and the synoviocytes secrete latent TGFβ into the synovial fluid, which becomes activated with joint motion, and accumulates in the superficial zone of articular cartilage [31]. Mechanical shearing within the joint activates latent TGFβ1 in the synovial fluid, rendering it available to stimulate TGFβ signaling in chondrocytes [32]. Though active TGFβ accumulates in the superficial zone, it is unable to penetrate deeper into middle and deep zones of cartilage [31]. In this way, superficial chondrocytes may receive adequate active TGFβ from synovial fluid to maintain homeostatic TGFβ signaling. In fact, physiologic dynamic compression of devitalized cartilage explants does not directly activate ECM-bound latent TGFβ in the deep zone of articular cartilage [33]. Therefore, activation of local TGFβ stores within the deep zone requires chondrocyte-dependent mechanisms. Taken together, articular cartilage relies upon synovial and cartilage-derived TGFβ, activated by mechanical and chondrocyte-dependent mechanisms, to sustain TGFβ signaling across the different articular cartilage zones.

Both physiologic and excessive loading of articular cartilage results in rapid induction of the canonical TGFβ signaling pathway [34]. In response to dynamic compression of either physiologic or excessive force, articular cartilage explants increase the mRNA levels for established TGFβ-inducible genes *Serpine1* and *Smad7*, and production of *Tgfb1* mRNA through an ALK5-dependent mechanism, demonstrating that TGFβ signaling and TGFβ production respond to cartilage loading [34]. The loss of compressive loading of cartilage explants results in a rapid decrease in TGFβ signaling, as shown by the reduction of pSMAD2 in chondrocytes and decreased expression of TGFβ-responsive genes *Serpine1*, *Smad7*, and *Alk5*, that is restored upon re-loading [35]. As a consequence of the unloading-dependent loss of TGFβ signaling, increased expression of *Col10a1* mRNA, a marker of chondrocyte hypertrophy, is observed, suggesting a TGFβ-dependent mechanism that couples joint loading to cartilage homeostasis [35].

In response to load in either cartilage or bone, relative *Alk5* expression increases, but the consequences of this change for joint homeostasis depend upon the cell compartment. In cartilage, loading reduces *Alk1* expression and increases *Alk5* expression to support canonical TGFβ signaling, unveiling one mechanism by which the balance between canonical and non-canonical TGFβ signaling in cartilage is preserved [34, 35]. In aging cartilage, the relative ratio of ALK1/ALK5 receptors is increased, thus favoring a non-canonical TGFβ signaling pathway [36, 37]. In response to either physiologic or excessive mechanical load, aged cartilage has a reduced ability to induce canonical TGFβ signaling, with reduced overall levels of phosphorylated SMAD2 and diminished nuclear localization of phosphorylated SMAD2 [38]. Thus, the ability of loading to sustain chondrocyte homeostasis may decline with age, and other avenues to promote cartilage health may become relevant, while canonical TGFβ signaling becomes less favored. Interestingly, perturbed joint loading due to transection of the anterior cruciate ligament (ACL) increases ALK5 in subchondral bone mesenchymal stem cells (MSCs) without increasing ALK1 [39]. The extent to which the MSC response to ACL injury reflects cellular exposure to excessive or diminished loads, or a cell type–specific response to altered joint loading, remains to be determined.

Subchondral bone, likewise, is dynamically loaded during joint motion and provides mechanical support to the overlying cartilage. The load-induced response of bone is largely mediated by osteocytes, the primary mechanosensors of bone [40]. Osteocytic TGFβ signaling is required for bone mechanosensation, as well as the control of bone quality [41, 42]. We found that increased loading on the articular cartilage and underlying subchondral bone, as a result of meniscal injury in mice, represses the expression of TβRII in subchondral bone plate osteocytes [43•]. Likewise, subchondral bone from mice with a genetic deficiency in osteocytic TβRII fails to respond to injury-induced load. Therefore, TGFβ signaling is both regulated by and required for osteocytic mechanosensitivity [43•]. In the same model of osteocytic TGFβ deficiency, articular cartilage deteriorates, suggesting that the mechanosensitive function of the subchondral bone plate requires osteocytic TGFβ signaling in order to maintain the health of cartilage [43•].

## Tissue-Specific Function of TGFβ Signaling in Joint Disease

TGFβ plays a tissue-specific role in joint homeostasis and joint disease, contributing to the sophisticated function of TGFβ in the joint (Table 2). For example, inhibiting TGFβ within the joint can prevent osteophyte formation but simultaneously exacerbate cartilage degeneration [44]. Tissue-level evidence in clinical specimens offers insight into the role of TGFβ in joint disease, and transgenic mouse models of joint disease with a tissue-specific ablation of key receptors or effectors within the TGFβ signaling pathway provide powerful tools for isolating the tissue- or cell-type–specific functions of TGFβ in joint disease.

**Table 2:** Tissue-specific differences of TGFβ signaling leading to OA

		Reference
Bone		
Excessive:	Increased *TGFB1* mRNA, TGFβ1 ligand, and *SMAD3* mRNA in human OA bone	[12–14]
	Increased *TGFB1* mRNA, TGFβ1 ligand, and *SMAD3* mRNA in primary OA osteoblasts	[45–49]
	Camurati-Engelmann disease with excessive levels of TGFβ1 secreted by osteoblasts develop OA	[39]
Repressed:	Loss of osteocytic TβRII leads to cartilage degeneration	[43•]
Cartilage		
Excessive:	Increased TGFβ1 and β2 ligands in human OA cartilage	[58]
	*SMAD3* mRNA higher in human OA cartilage	[16]
Repressed:	Loss of chondrocyte-intrinsic SMAD3, TβRII, or ALK5 in mice induces OA	[71–73]
	Downregulation of TGFβ ligand, reduced TGFβ-induced proteoglycan synthesis, and decreased TβRI and TβRII in aging cartilage	[85, 91]
Altered canonical/non-canonical TGFβ signaling:	Increased ALK1/ALK5 receptor ratio increases MMP13 in OA cartilage	[36]
	TβRI competitively antagonizes BMP signaling	[67•]
	SMAD1/5/8 signaling promotes hypertrophic chondrocyte differentiation during chondrogenesis	[74]
	Increased ALK1/ALK5 receptor ratio in aging murine and bovine cartilage	[36, 37]
Synovium		
Excessive:	Increased TGFβ ligand in synovial fluid induces OA	[17, 64–66]
Repressed:	Reduced TGFβ signaling due to dominant-negative mutation of TβRII exhibit synovial hyperplasia	[68]
	Synovial fluid TGFβ ligand antagonizes degenerative effects of IL-1 on cartilage	[87, 88]
Mesenchymal stem cells		
Excessive:	Excessive TGFβ signaling in MSCs after injury results in OA	[39, 89]
Repressed:	Higher concentrations of TβRI inhibitor induce proteoglycan loss in cartilage	[39]

### Bone-Intrinsic Role of TGFβ in Joint Health and Disease

The association of altered bone mass and mineralization with OA progression [1], coupled with differences in expression of genes critical for TGFβ signaling in OA bone [12–14], has led to an interest in understanding the role of TGFβ in bone during joint degeneration. Disrupted TGFβ signaling may participate in several possible mechanisms by which bone may contribute to cartilage degeneration in joint disease. These mechanisms include altering joint mechanics, disrupting nutrient and vasculature exposure through the osteocytic lacunocanalicular network, or changing levels of paracrine factors that disrupt cartilage health. All three bone-cell types — osteoblasts, osteoclasts, and osteocytes — are distinctly regulated by TGFβ signaling in joint health and disease.

Studies of TGFβ signaling in whole-bone samples from OA human joints have found increased TGFβ ligand production and *TGFB1* and *SMAD3* gene expression, relative to non-arthritic joints, even in bone specimens taken from sites distant from the OA joint [12–14]. Microarray gene expression of bone from OA patients demonstrates significant increases in expression of *SMAD3* [12]. These disruptions in TGFβ signaling occur in the context of altered WNT/β-catenin signaling [12, 14]. Taken together, the differential gene expression patterns in OA bone are consistent with perturbed osteoblast differentiation, altered bone formation, and heterogenous mineralization. Given the mixture of bone-cell types within whole bone, it is necessary to further delineate the specific contributions of each cell type to TGFβ signaling in joint homeostasis to better characterize the bone-intrinsic role.

The evaluation of primary osteoblasts isolated from human OA specimens supports an osteoblast-intrinsic role of TGFβ in OA. Similar to findings in whole bone extracts, primary osteoblasts obtained from OA bone express increased levels of *TGFB1* mRNA, TGFβ1 ligand, and *SMAD3* mRNA [45–49]. OA osteoblasts exhibit a TGFβ-dependent shift from the normal ratio of *COL1A1/COL1A2* mRNA of approximately 2:1 to levels 2–threefold higher than that in normal osteoblasts, which blunts the ability of osteoblasts to generate a mineralized ECM in vitro [15, 47]. When TGFβ1 is inhibited, OA osteoblasts correct the abnormal *COL1A1/COL1A2* ratio at the mRNA level and mineralize normally [47]. In murine osteoblasts, SMAD3 represses the function of RUNX2 [50], a key regulator of osteoblast differentiation and mineralization. Increased *SMAD3* in human OA osteoblasts is associated with the calcium:phosphorous ratio in bone mineral [45], likely through its regulation of RUNX2-dependent mineralization.

TGFβ signaling in OA osteoblasts exhibits an epistatic relationship with the Wnt signaling pathway, an important regulatory pathway in bone formation [45, 48, 49]. TGFβ antagonizes the Wnt signaling pathway in OA osteoblasts by increasing the mRNA expression of Dickkopf-2 (*DKK2*), which diminishes osteoblastic mineralization in response to BMP-2 [48]. Increased hepatocyte growth factor (*HGF*) mRNA and HGF protein in OA osteoblasts increase *TGFB1* expression, which subsequently inhibits the Wnt signaling pathway and blunts BMP-2-dependent mineralization [49]. The loss of HGF, therefore, favors BMP-2 signaling through SMAD1/5/8 and stimulates the canonical Wnt signaling pathway [49].

Camurati-Engelmann disease (CED) is a disease of poor bone quality due to excessive levels of TGFβ1. A mouse model of CED, in which the *Col1A1* promotor drives expression of *Tgfb1* with the point mutation H222D, results in higher levels of active TGFβ1 secreted by osteoblasts [39]. Coupled with the disrupted subchondral bone, these mice develop thin articular cartilage with relative hypocellularity, thick calcified cartilage, and excessive subchondral bone vascularity [39]. The subchondral bone in these mice exhibits increased levels of nestin-positive MSCs and osterix-positive osteoprogenitors, suggesting that excessive TGFβ signaling alters the joint environment to increase recruitment of MSCs. This, in turn, increases the number of available osteoprogenitors [39]. This MSC-dependent mechanism illustrates how excessive TGFβ secreted by subchondral bone osteoblasts can exacerbate OA.

On the other hand, inadequate subchondral bone TGFβ signaling also has negative consequences for joint homeostasis. TGFβ signaling in osteocytes is essential for function of these cells, regulating their ability to maintain the lacunocanalicular network through osteocytic perilacunar/canalicular remodeling (PLR) [41, 42]. In addition to their role in maintaining bone quality, osteocytes have recently emerged as cellular contributors to OA [43•, 51•]. More specifically, osteocytic TGFβ signaling is required for the response of subchondral bone to joint injury [43•]. Without this key signaling pathway, subchondral bone homeostasis is disturbed, resulting in arthritic cartilage degeneration [43•].

As mediators of bone homeostasis, osteoclasts are inevitably involved in joint health. The altered mass and mineralization in OA subchondral bone suggest an imbalance in bone remodeling by osteoblasts and osteoclasts, for which TGFβ is a critical coupling factor [52]. Osteoclasts can play a direct or indirect role on the effect of TGFβ within the joint. Directly, osteoclasts require TGFβ for osteoclastogenesis. Osteoclasts also locally control the level of active TGFβ by liberating and activating latent TGFβ from the bone matrix [53–55]. In an ACLT model of OA, osteoclast suppression with alendronate reduces local TGFβ activation, with a corresponding reduction in matrix metalloproteinase 13 (MMP13) in articular cartilage and osteophyte formation [56]. Indirectly, osteoclasts are sensitive to aberrant TGFβ signaling within the joint because it can alter *Rankl* expression by osteocytes and interfere with osteoblast-osteoclast coupling during bone remodeling [41, 57]. Therefore, the activity of osteoclasts can impact the level of active TGFβ signaling, and vice versa, with broader impacts throughout the joint.

### Cartilage-Specific Role of TGFβ in Joint Health and Disease

Human osteoarthritic cartilage shows a complex disruption of homeostatic TGFβ signaling at various levels involving ligand, receptor, effector, and negative regulators, underscoring the importance of studying the hierarchy of altered TGFβ signaling in OA. There are at least three ways that altered TGFβ signaling in human cartilage is implicated in OA. First, excessive activity of the pathway is observed in human OA cartilage: the expression of genes encoding TGFβ1 and β2 ligands is positively correlated with cartilage degeneration [58]. Likewise, *SMAD3* mRNA levels are significantly higher in OA cartilage, in a manner that is unexplained by DNA methylation in the *SMAD3* promoter region [16]. Second, there is a shift from canonical to non-canonical signaling: the mRNA expression of *ALK1* in human OA cartilage is correlated with chondrocytic *MMP13* mRNA expression, whereas *ALK5* mRNA correlates with aggrecan and type II collagen mRNA expression. Since ALK1 preferentially activates SMAD1/5/8, and ALK5 preferentially activates SMAD2/3, this suggests a role for TGFβ receptor and effector selection in expression of genes that are protective or deleterious for cartilage [36]. Third, there is aberrant negative regulation of the signaling pathway: in human OA cartilage, higher levels of SMURF2, a multi-level negative regulator of TGFβ signaling, can be observed compared to healthy cartilage [59]. SMURF2 ubiquitinates SMADs and the TβRI receptor to promote degradation and reduce TGFβ signaling. Therefore, high levels of SMURF2 indicate a general reduction in TGFβ signaling.

In addition to direct regulation of the signaling pathway, TGFβ signaling can regulate, and be regulated by, microRNAs (miRNAs) to affect downstream signaling and OA gene expression. The expression of miR-140, for instance, is repressed by TGFβ signaling and significantly reduced in human OA cartilage [60–62], whereas miR-455, which promotes TGFβ/SMAD3 signaling, is also repressed in OA cartilage [63]. Overall, these observations demonstrate an association of cartilage degeneration with aberrant TGFβ signaling, whether by suppressed or excessive activity, a shift from canonical to non-canonical signaling, or altered negative regulation. While these associations in human cartilage from end-stage OA show clinical relevance and motivate further study of TGFβ signaling in chondrocytes, other approaches are needed to establish causal mechanisms that operate earlier in the disease process.

Findings in animal models reinforce the importance of an optimal level and type of TGFβ signaling in the joint. Increased TGFβ in the synovial fluid, whether by intra-articular administration of TGFβ or overexpression of TGFβ by the synovium, induces cartilage degeneration and osteophyte formation [64–66]. Elevated TGFβ signaling activates a positive feedback loop that further increases expression of *Tgfb1*, *b2*, and *b3* mRNAs in the synovium and cartilage, relative to vehicle-injected controls [64–66]. Conversely, inhibition of endogenous TGFβ by systemic injection of scavenging soluble TβRII prevents osteophyte formation but, likewise, induces proteoglycan degeneration in cartilage [44]. At the chondrocyte-level, although the expression of both receptors declines with age, the number of cells positive for ALK5 in OA cartilage decreases more rapidly than those positive for ALK1. This consequently increases the ALK1/ALK5 ratio and shifts the function from ALK5-dependent collagen II and aggrecan expression and, instead, favoring SMAD1/5/8 signaling and MMP13 production [36]. In cartilage development at the growth plate, TβRI competitively antagonizes BMP signaling mediated by ALK1, thus shifting the balance of TGFβ signaling [67•]. Together, these results highlight the importance of an optimal quantity and quality of TGFβ signaling, since excessive or inhibited TGFβ activity, or a shift in the balance of canonical and non-canonical TGFβ signaling, can exacerbate cartilage degeneration.

Transgenic mouse models with disrupted TGFβ signaling in multiple tissues have likewise demonstrated the requirement of TGFβ signaling in cartilage health. Mice with a dominant-negative mutation of the gene encoding TβRII in the articular cartilage, synovium, periosteum, and perichondrium exhibit a severe OA phenotype with substantial cartilage loss and osteophyte formation [68]. This mutation suppresses the responsiveness to TGFβ ligand, thus promoting terminal differentiation of chondrocytes, reducing proteoglycan synthesis, and increasing type X collagen [68]. Mice with a global loss of canonical TGFβ effector SMAD3 similarly develop severe OA [69, 70]. Taken together, these two mouse models illustrate the chondroprotective role of TGFβ in synovial joint tissues, motivating mechanistic study of the tissue-specific effects of TGFβ in articular cartilage.

Because OA involves the coordinated disruption of multiple joint tissues, mouse models employing chondrocyte-intrinsic mutations in the TGFβ signaling pathway can diminish the apparently confounding effects of TGFβ signaling in joint crosstalk. Toggling this pathway experimentally by targeting the receptors, effectors, and negative regulators in chondrocyte-intrinsic models, for example, by driving transgenic expression of Cre recombinase or other genes using a *Col2a1* promoter, isolates the relative chondroprotective nature of TGFβ. As mentioned previously, SMURF2 levels are elevated in human OA cartilage, disrupting homeostatic TGFβ signaling [59]. Mice with a chondrocyte-intrinsic overexpression of SMURF2 develop spontaneous OA with loss of articular cartilage, increased subchondral bone sclerosis, and increased mRNA expression of type X collagen and elevated levels of MMP13 protein in the articular cartilage [59], demonstrating the chondrocyte-intrinsic requirement of balanced TGFβ signaling. Likewise, mice with a chondrocyte-intrinsic loss of *Smad3* develop OA and demonstrate decreased expression of genes encoding aggrecan and collagen II associated with increased MMP13 protein levels and enzyme activity in the articular cartilage, thus indicating that chondrocyte-intrinsic SMAD3 is essential for maintenance of healthy cartilage [71]. At the receptor level, chondrocyte-specific ablation of the gene encoding either TβRII [72] or ALK5 [73] promotes joint degeneration by inducing genes implicated in cartilage destruction. More specifically, the deletion of *Tgfbr2* in chondrocytes exacerbates OA and induces *Runx2*, *Mmp13*, and *Adamts5* gene expression in articular cartilage [72]. Interestingly, the deleterious effects of chondrocytic *Tgfbr2* ablation were mediated by activities of proteinases encoded by *Mmp13* and *Adamts5*, since deletion of either gene or treatment with an MMP13 inhibitor ameliorated the OA phenotype in this model [72]. Likewise, in chondrocyte culture, siRNA-mediated inhibition of TβRII upregulated *Mmp13* and *Col10a1* gene expression through a RUNX2-dependent mechanism [72]. Furthermore, chondrocyte-intrinsic deletion of *Alk5* resulted in a spontaneous OA phenotype with articular cartilage degradation, synovial hyperplasia, osteophyte formation, and subchondral bone sclerosis, with reduced SMAD3 phosphorylation and increased *Mmp13*, *Adamts5*, and *Col10a1* gene expression and elevated protein levels of MMP13 and ADAMTS5 [73].

Some of the effects of TGFβ signaling in cartilage are explained by its ability to control chondrocyte differentiation. Prior to chondrogenesis, MSCs require both ALK5-dependent SMAD2/3 activity and ALK1-dependent SMAD1/5/8 activity, in concert with SMAD4, to induce *COL2AI* gene expression and subsequently produce increased protein levels of collagen II [74–76]. As chondrogenesis proceeds, the function of SMAD1/5/8 shifts to promote terminal hypertrophic chondrocyte differentiation, such that it inhibits collagen II production and increases MMP13, collagen X, and alkaline phosphatase [74]. On the other hand, SMAD3 acts to expand the pool of chondrocytes by stimulating their differentiation and preventing their hypertrophy [77–79]. In MSCs, TGFβ increases the transcriptional activity of SOX9 and the gene expression of *COL2A1* [78]. Through SMAD2/3-dependent signaling, TGFβ inhibits chondrocyte hypertrophy [77, 78] and decreases expression of the genes encoding collagen X and other terminal differentiation markers [79]. TGFβ tightly regulates its own signaling in chondrocytes, both by positive feedback that increases the expression of TGFβ receptors and SMAD3 [80] and by negative feedback that can reduce mRNA stability of TGFβ receptors, decrease SMAD3, and increase SMAD7 [80]. Decreased levels of TGFβ signaling shift chondrocytes from expression of chondroprotective genes such as *SOX9* and *COL2A1* to hypertrophic genes such as *COL10A1* [80]. In addition to the availability of TGFβ ligand and the relative balance of SMAD2/3 signaling, the chondrogenic response to TGFβ depends on mechanical cues from the ECM [30], as mentioned previously.

Among the constituents of the TGFβ pathway that control chondrocyte homeostasis, the most well studied is SMAD3, motivated by the association of human OA and *SMAD3* variants identified in GWAS [19–21]. In chondrocytes, SMAD3-dependent TGFβ signaling promotes cartilage health by repressing RUNX2-inducible *Mmp13* and *Col10* gene expression and tempering BMP signaling [71, 72, 81], each through mechanisms that diminish non-canonical TGFβ signaling. Chondrocytes exhibit a time-dependent response to exogenous TGFβ, with an early repression of *Mmp13* mRNA and MMP13 protein requiring SMAD3 and a later induction of *Mmp13* mRNA and MMP13 protein requiring p38 [71]. Importantly, in the absence of SMAD3, non-canonical TGFβ signaling through p38 is favored, inducing the cartilage-destructive enzyme MMP13 [71]. Primary chondrocytes from mice lacking SMAD3 show enhanced BMP-related gene expression by increasing *Smad1*, *Smad5*, *BMP2*, and *BMP6* mRNA levels, resulting in increased phosphorylated SMAD1/5/8 and *Col10* mRNA expression and accelerating chondrocyte maturation [81]. This aberrant signaling can be blocked by overexpressing *Smad2* and *Smad3* or inhibiting BMP signaling [81]. TGFβ likewise counteracts the effects of MMPs and protects the local cartilage ECM by inducing expression of tissue inhibitor of metalloproteinases-3 (TIMP-3) through SMAD2/3-dependent and ERK1/2-dependent mechanisms [82, 83], illustrating the multiple avenues by which SMAD3 functions to promote chondrocyte homeostasis.

### TGFβ and Synovial Inflammation

As in cartilage, a balanced level of TGFβ signaling in the synovium and synovial fluid is required for healthy joint homeostasis, with insufficient or excessive TGFβ levels compromising joint health. The ability of TGFβ to induce a suppressive or inflammatory immune response within the synovium is context-dependent [84]. The synovium is a source of TGFβ ligand within the joint [32, 85], and the level of TGFβ in synovial fluid correlates positively with OA severity [17]. TGFβ is also a key mediator of synovial hyperplasia in rheumatoid arthritis [86]. Either excessive or suppressed TGFβ in the synovium or the synovial fluid can result in synovial hyperplasia. For instance, mice with reduced TGFβ signaling throughout the joint due to a dominant-negative mutation of the gene encoding TβRII in multiple joint tissues exhibit synovial hyperplasia [68]. Excessive levels of TGFβ within the joint, as a result of intra-articular injection of exogenous TGFβ, likewise promote synovial hyperplasia [64]. On the other hand, synovial inflammation with joint degeneration involves production of inflammatory cytokines, such as interleukin (IL)-1. TGFβ antagonizes the degenerative effects of IL-1 on cartilage [87, 88]. Together, TGFβ and the synovium can participate in bidirectional feedback, where synovial production of TGFβ can have broader effects on other joint tissues, and TGFβ signaling in other joint tissues can affect the synovium.

### Mesenchymal Stem Cells and TGFβ in Joint Health and Disease

In addition to the role of subchondral bone osteoblasts, osteoclasts, and osteocytes in synovial joint homeostasis, there is evidence of an influx of bone marrow MSCs into the subchondral bone during OA progression, which induce angiogenesis and differentiate into osteoblasts to further perturb the subchondral bone environment [39]. Joint injury from ACL transection increases the level of nestin-positive MSCs in the subchondral bone marrow and osteoprogenitor clusters in the bone marrow [39, 89]. Systemic administration of a TβRI inhibitor can reduce the number of MSCs and osteoprogenitors, normalize the subchondral bone, and attenuate OA after injury [39]. The benefits of the TβRI inhibitor on joint health are tissue-dependent; higher concentrations of the TβRI inhibitor not only mitigate injury-induced changes in subchondral bone structure but also induce proteoglycan loss in cartilage, underscoring the tight control of TGFβ that is necessary for joint health [39]. The relative benefits of inhibiting TGFβ signaling within the subchondral compartment are mediated by nestin-positive MSCs. A nestin-positive MSC-specific ablation of the gene encoding TβRII recapitulates findings with the pharmacologic TβRI inhibitor, with protection of subchondral bone microarchitecture and cartilage proteoglycan levels after ACL transection [39].

With increased levels of TGFβ in circulation after injury, targeting the TGFβ signaling pathway at the level of the ligand, rather than receptor, could likewise normalize the aberrant environment after injury. Local treatment to the subchondral bone or systemic treatment with an inhibitor of TGFβ1, β2, and β3 reduces the number and mobilization of nestin-positive MSCs in the subchondral bone and diminishes angiogenesis after ACL transection [39, 89]. This effect is dose-dependent, such that lower and higher concentrations lead to proteoglycan loss [89]. As discussed below, when considering the benefits of inhibiting excess TGFβ signaling post-injury, care must be taken not to compromise the essential role of this growth factor in other joint cell types where it also supports joint homeostasis.

## TGFβ in Aging and OA

Aging can have broad effects on TGFβ signaling in multiple joint tissues. Because the prevalence of OA increases with age, considering the shift in function of TGFβ with age could identify a distinct age-related pathophysiology of cartilage degeneration. Furthermore, studying spontaneous age-related OA allows for the evaluation of joint degeneration in the absence of the substantial mechanical and inflammatory changes that occur with joint injury in post-traumatic OA models.

Among the many roles of TGFβ in the joint is its interaction with IL-1, a pro-inflammatory cartilage destructive cytokine that is upregulated with age and following injury [90]. TGFβ and IL-1 interact, such that TGFβ can protect against the deleterious effects of IL-1 on proteoglycan synthesis [87, 88]. With age, however, IL-1-induced cartilage degeneration outpaces the protective effects of TGFβ [87, 88, 91], mediated in part by nitric oxide production [88]. While TGFβ blocks IL-1-induced nitric oxide production in young mice, TGFβ is unable to induce the same response in old mice, likely due to decreased expression of TGFβ receptors [88]. Interestingly, while TGFβ shows protective effects on cartilage synthesis in the presence of IL-1, it exacerbates the inflammatory response, generating a severe synovitis, underscoring the importance of delineating the tissue-specific effects of TGFβ [87]. A similar role for IL-1 and TGFβ crosstalk has been shown in equine cartilage, where TGFβ-induced proteoglycan synthesis is diminished by the addition of IL-1 [92].

Aging cartilage exhibits decreased levels of TGFβ1, β2, and β3 ligands, diminished TGFβ-induced proteoglycan synthesis, and reduced the number of cells positive for TβRI and TβRII protein [85, 91]. This age-related suppression of TGFβ ligands and receptors results in decreased phosphorylated SMAD2 without a reduction in overall SMAD2 expression, suggesting reduced active canonical TGFβ signaling [91]. Furthermore, a mouse model of spontaneous OA demonstrated lower levels of TGFβ3 ligand and phosphorylated SMAD2 over the course of OA progression with a complete loss by 1 year of age, coinciding with an increase in protein levels of BMP-2 with age [85].

Across species, articular cartilage exhibits a shift from canonical to non-canonical TGFβ signaling during aging [36, 37, 93]. In aging murine cartilage, increased ALK1/ALK5 ratio favors TGFβ-induced SMAD1/5/8 signaling and MMP13 production [36]. A similar increase in the ALK1/ALK5 ratio is observed in aged bovine cartilage [37], and aged chondrocytes from guinea pigs demonstrate a progressive shift of SMAD2/3 signaling to SMAD1/5/8 signaling [93], illustrating the age-related shift in TGFβ function across multiple species. Aged bovine cartilage demonstrates reduced activation of SMAD2/3 signaling and nuclear localization in response to either stimulation with TGFβ or mechanical activation [38]. These changes with age precede gross degeneration of cartilage and, therefore, may be early signs of OA [38]. Together, these findings emphasize the importance of the relative balance of canonical and non-canonical TGFβ signaling in cartilage health.

## Interactions of TGFβ and Other Joint Tissues

Aging also impacts the TGFβ-induced collagen production in ligamental fibroblasts. With aging, the ability of medial collateral ligament (MCL)-derived fibroblasts to synthesize collagen in response to TGFβ is diminished relative to young controls [94]. While overall collagen synthesis decreases at all doses in MCL-derived fibroblasts from older rabbits, the sensitization to TGFβ is higher in aged animals, such that relative to controls lacking TGFβ, collagen synthesis increases with increasing doses of TGFβ [94]. These findings suggest that the diminished mechanical integrity and prolonged healing with injury of aged ligaments may be due to a weakened response to TGFβ. Coupled with the increased sensitization to TGFβ, treatment with TGFβ could, thus, improve the mechanical stability of the ligament.

One area of interest in the intersection of TGFβ and joint homeostasis is the extent of crosstalk with the nervous system. Nerve growth factor (NGF) is a key driver of the musculoskeletal pain response. Despite the lack of apparent sequence similarities, TGFβ and NGF demonstrate similarities in topological structure that place them in a common growth factor superfamily [5]. Anti-NGF therapies in OA reduce knee OA pain [95] but increase the incidence of rapidly progressive OA [96]. Therefore, understanding the extent to which TGFβ is involved in joint pain could provide further context for the complexity of targeting NGF.

## Targeting TGFβ for Treatment of Joint Disease

Careful consideration must be taken when developing TGFβ-targeting treatments for OA due to the complex role of TGFβ in joint health and disease. Treatment strategies need exquisite control of multiple factors, including optimal dose, tissue-specific effects, selection of downstream TGFβ receptors and effectors, and local mechanical cues. For instance, injury can induce excessive levels of TGFβ ligand within the joint [39], suggesting that returning TGFβ to pre-injury levels would encourage joint health. However, injury also represses osteocytic TβRII in the subchondral bone, and reduced levels of TGFβ ligand may further reduce downstream osteocytic TGFβ signaling [43•]. Furthermore, in aging cartilage, the relative balance of canonical and non-canonical TGFβ signaling is disrupted [36, 37, 93], complicating the efforts of targeting this pathway by enhancing or inhibiting TGFβ.

Therapeutics have been developed that target different levels of the TGFβ signaling pathway in the clinical setting, primarily in the context of cancer or fibrotic disease [97, 98]. Recently, human chondrocytes virally transduced with a gene containing TGFβ1 have been employed in the setting of patients with OA in a phase II clinical trial. Patients with knee OA received an intra-articular injection of either placebo or transduced allogenic chondrocytes expressing TGFβ1, which significantly improved clinical pain scores [99, 100]. Further work is needed to fully uncover the relative benefit of delivering TGFβ1-producing chondrocytes, designed to be a cell-mediated cytokine gene therapy. Another critical consideration is the likelihood that OA originates through several distinct mechanisms, each of which may respond differently to changes in the level or type of TGFβ signaling. Overall, the complex nature of the TGFβ signaling pathway suggests that therapeutically regulating TGFβ would require a precise understanding of the underlying etiology of OA and the specific disruption in TGFβ to successfully use it as a therapeutic target of OA.

## Conclusion

In conclusion, TGFβ signaling plays a sophisticated function in maintaining healthy joint crosstalk that is non-linear and depends upon effector selection, physical and mechanical cues, and tissue-specific function to support joint health. Synovial joints facilitate smooth motion and load transfer through integrated function of multiple tissues, including articular cartilage, bone, ligaments, tendons, synovium, and menisci. Under normal conditions, the biological and mechanical activities of these tissues are exquisitely coordinated, yet in the setting of aberrant TGFβ signaling, one or more of these joint tissues can deteriorate leading to overall joint destruction. Regulation of the TGFβ signaling pathway can occur at different levels, and disrupting the homeostatic TGFβ signaling at any level of the pathway can have broad effects through crosstalk among multiple joint tissues.
